# Developing novel smoking cessation resources for Aboriginal people who smoke tobacco and their healthcare providers: a mixed methods study

**DOI:** 10.1186/s12913-025-13568-x

**Published:** 2025-11-28

**Authors:** Kelsey Sharrad, Sasha Stewart, Nick Marlow, Alyson J. Crozier, Ian Gwilt, Adrian Esterman, Gillian Gould, Antony Veale, Alwin Chong, Haydyn Bromley, Kristin Carson-Chahhoud

**Affiliations:** 1 Adelaide Medical School, University of Adelaide the University of South Australia, Adelaide, Australia; 2 Allied Health and Human Performance, University of South Australia, Adelaide, Australia; 3 Rosemary Bryant AO Research Centre, Clinical and Health Sciences, University of South Australia, Adelaide, Australia; 4 Alliance for Research in Exercise, Nutrition and Activity, University of South Australia, Adelaide, Australia; 5 UniSA Creative, University of South Australia, Adelaide, Australia; 6 Faculty of Health, Southern Cross University, Lismore, New South Wales Australia; 7 Respiratory Medicine Unit, The Queen Elizabeth Hospital, Adelaide, South Australia Australia; 8 Lowitja Institute, Carlton, Victoria Australia; 9 Bookabee Australia, Adelaide, South Australia Australia

**Keywords:** Aboriginal health, Smoking cessation, Tobacco, Digital health, Augmented reality, Qualitative research

## Abstract

**Introduction:**

Many health professionals (HPs) are reluctant to offer smoking cessation advice to Aboriginal Australians who smoke; suggesting a need for better education to improve HP skills and knowledge to increase confidence. Previous studies exploring culturally relevant smoking cessation training for HPs have been effective among HPs working with Aboriginal Australians. Thus, identifying mechanisms to increase accessibility of these programs is an important public health issue. Augmented reality (AR) has the potential to enhance digital health interventions, and is effective and acceptable across a range of fields. Under the guidance of an Aboriginal advisory group, two sets of resources were developed; i) patient education and cessation resources for use by Aboriginal people who smoke, and ii) HP resources providing brief education to support Aboriginal patients who smoke. The aim of this study was to explore the acceptability of, and iteratively co-design these resources with HPs through qualitative interviews and questionnaires.

**Methods:**

HPs were recruited from two South Australian public hospitals between August 2020 and June 2021. One-on-one interviews were conducted with n=18 HPs, with transcripts coded using the Theoretical Framework of Acceptability (TFA).

**Results:**

Participants found that the AR-embedded patient resources provided engaging and relatable quit smoking support. The HP resources based on brief counselling techniques were considered a helpful reminder to prompt a smoking cessation conversation with Aboriginal patients.

**Conclusion:**

These resources were considered acceptable by HPs who treat Aboriginal Australians who smoke, and may serve as a useful adjunct to HP training sessions.

**Supplementary Information:**

The online version contains supplementary material available at 10.1186/s12913-025-13568-x.

## Introduction

Aboriginal and Torres Strait Islander Australians continue to experience poorer health and have a shorter life expectancy than non-Aboriginal Australians [1]. Smoking is a leading risk factor for the most common causes of death among Aboriginal people (cancer and circulatory diseases [2]), who are almost three times as likely to smoke, with a daily [3] prevalence rate of 41% compared with 13% among non-Aboriginal Australians [4]. This is despite decreasing prevalence rates for both the general Australian population (6.8% decrease between 2004 and 05 and 2014-15), and Aboriginal Australians (8.6% decrease between 2004 and 05 and 2014-15) [5]. This suggests that Aboriginal and non-Aboriginal people who smoke have not equally benefitted from the policies and interventions that have been offered in Australia throughout the last 40 years [6]. This is unsurprising, given the profound disadvantage experienced by this group as a result of harmful government policies enacted since colonisation [7], which has contributed to issues of systemic racism within Australian society as well as inappropriate healthcare and research methodologies [8]. Following recommendations by the advisory group overseeing this study, the term “Aboriginal” will henceforth be used to respectfully refer to the Indigenous population, with our acknowledgement of the diversity within Aboriginal communities.

Health professionals (HPs) play an important role in encouraging and supporting their patients to quit smoking, with even brief intervention shown to be beneficial in increasing rates of cessation [9, 10]. Unfortunately, HPs report that they are often unable to provide this advice to their patients who smoke [11–13], with identified barriers including lack of time, knowledge, skills, and confidence [14, 15]. Furthermore, research shows that many HPs are reluctant to offer smoking cessation advice to Aboriginal people who smoke in particular, due to both a lack of confidence in the necessary cultural sensitivity and communication skills [15], and, in pregnant women particularly, a perceived low probability of success [16]. However, Cochrane systematic reviews have shown that brief HP training can address the aforementioned issues in both primary care [12] and community pharmacy [17] settings; suggesting a need for better training and education to improve HP skills and knowledge to increase confidence and, ultimately, the likelihood that they will offer evidence-based smoking cessation advice. Previous studies exploring culturally relevant smoking cessation training for HPs have been effective in increasing skills and confidence among HPs working with Aboriginal Australians [9]. Therefore, identifying mechanisms to increase accessibility of training programs is an important public health issue.

As smartphone use in Australia continues to rise [18] – particularly among Aboriginal youth [19] - the role of technology in future public health campaigns becomes increasingly apparent. One such technology which shows promise for health interventions is augmented reality (AR). AR is an interactive technology whereby digital objects appear to exist within the real world [20]. Benefits of utilising this interactive technology to deliver health information include reducing inequalities associated with poor health literacy [21], geographic reach and accessibility of information [22], and the ability to tailor resources to individual population groups based on cultural guidelines or language [23, 24]. Systematic reviews demonstrate that digital interventions such as AR may be beneficial for smoking cessation [25, 26]; including the use of AR imagery for cue exposure therapy [27], and AR anti-smoking imagery overlaid on the bodies of people who smoke [28].

Under the guidance of an Aboriginal advisory group along with experts in respiratory health, smoking cessation, and AR technology, two sets of resources were developed; (i) patient education and cessation resources for use by Aboriginal people who smoke, and (ii) HP resources which provide brief education to improve skills and knowledge to support Aboriginal patients to quit smoking. The aim of this study was to explore the acceptability of these resources by HPs through qualitative interviews, and to iteratively co-design a final version of resources for future feasibility and efficacy testing [29, 30].

## Methods

### Study design

A mixed methods triangulation research design [31] was utilised to explore the acceptability of resources by HPs, while the iterative co-design process was guided by the Plan, Do, Study, Act (PDSA) cycle framework. PDSA allows for the timely improvement and adaptation of an intervention based on issues identified through end-user feedback [32]. Evidence suggests that smoking cessation interventions that are culturally appropriate and tailored to the target population are most likely to be successful [33]; the printed materials and associated digital content were therefore developed under the guidance of an Aboriginal advisory group, together with a group of experts in the areas of AR technology and design, behaviour change, Aboriginal health, smoking cessation, and respiratory health. Through the present study, the iterative co-design process involved three rounds of interviews with different participants, with revisions made to resources following each; resulting in V1, V2 and V3 resources [29, 30]. To ensure the safe and appropriate conduct and reporting of research within the Aboriginal health space, this research was conducted in accordance with the CONSIDER statement [34], as per Appendix 1.

The patient resources were designed to convey best-practice smoking cessation strategies and education using culturally-tailored content and language. The HP resources included information on the three-step ‘Ask, Advise, Help’ smoking cessation counselling technique, developed by Quit Victoria, and considered best practice by the Royal Australian College of General Practitioners (RACGP) [35]. This counselling technique is designed to support all people to quit smoking, regardless of their level of motivation, using a non-confrontational approach and requiring less than 30 s [10], making it ideal for time-poor HPs. HP resources also included potential barriers to smoking cessation in the Aboriginal population, and appropriate strategies to aid support. The resources were intended to be delivered with AR using a bespoke smartphone application. Digital interactive information was superimposed over (i) a smoking cessation education workbook (for patients), and (ii) a postcard-sized information card (for HPs). Acceptability and usability of the resources were explored through three rounds of one-on-one semi-structured interviews.

### Study setting/recruitment

From August 2020 to June 2021, purposive and snowballing sampling methods were used to recruit the targeted *n* = 16 HPs working at two participating South Australian tertiary public hospitals – The Queen Elizabeth Hospital, and The Royal Adelaide Hospital. Ethnicity was not an inclusion or exclusion criteria for this project, and HPs were not asked to disclose their ethnicity in the demographic questionnaire (see Limitations within the Discussion).

Inclusion criteria:

- Either a: specialist medical officer, nurse, Allied Health Professional, trainee medical officer (e.g. Advanced Trainees and Registrars).
- Practicing health professional in South Australia.
- Able to provide written informed consent.
- > 18 years of age.
- Worked consistently over the previous 12 months with Aboriginal South Australians.

Exclusion Criteria:

- Not a resident of South Australia.

Unable to provide written informed consent.

HPs were recruited through flyers displayed in wards and offices of the participating hospitals, and researchers sought assistance from clinician contacts within their professional networks. Of those who saw the flyers, initial contact with the research team was made via email or phone call. Potential participants were screened to ensure they met inclusion criteria, and eligible participants were invited to schedule an interview at an appropriate time. A participant information sheet and consent form were emailed to the participant for their review. They were asked to bring the consent form to the interview to discuss further, and have it signed and witnessed by a member of the research team.

### Data collection

Following completion of a baseline questionnaire (collecting demographics along with baseline confidence, knowledge, and attitude levels), a semi-structured guide was used to explore HPs awareness of, and experience with, existing smoking cessation resources (targeting either people who smoke and/or HPs), willingness to use various types of resources, associated barriers and facilitators, preferences for delivery of the information, and anticipated effectiveness of the AR resources. Researchers then demonstrated functionality of the printed and AR resources, developed for (i) Aboriginal people who smoke and ii) HPs supporting Aboriginal people who smoke. Participants were then asked to view and interact with the resources. The second part of the interview explored the acceptability of the resources based on the participant’s experience. At each stage of the interview, the duration spent on each of the semi-structured questions was untimed to allow for participants to elaborate on any of the questions or to explore other aspects within the scope of the research topic. The interviewer also asked additional questions as required to clarify answers or seek additional information. The interviews were audio recorded and transcribed via an online AI transcription service.

Following the interview, participants completed another questionnaire (Appendix 2); including 7-point Likert scales exploring opinions on the AR resources (to assist with data triangulation [31]), and a software review which was developed using the Enlight protocol [36] – a tool used in the evaluation of digital health interventions.

Three rounds of interviews were undertaken with different participants, with revisions made to resources following each; resulting in V1, V2 and V3 resources. The first set of HP resources (V1) were postcards with digital AR information embedded; accessed by smartphone. V2 and V3 versions shown to participants were printed materials only (a penholder (V2) and poster (V3), each having the same content displayed in a different style) (see Appendix 3).

### Data analysis

The interview transcripts were analysed using the Theoretical Framework of Acceptability (TFA), developed by Sekhon et al. [37] to improve the robustness of acceptability research, demonstrated in previous research in health promotion [37, 38]. An important feature of this framework is that it enables assessment of a broad range of contextual factors; the TFA comprises 7 constructs incorporating both anticipated and experienced perceptions of and attitudes towards the intervention [37, 39]. The 7 constructs are affective attitude, perceived effectiveness, opportunity cost, burden, ethicality, self-efficacy, and intervention coherence. A codebook was developed based on these TFA constructs and emergent themes relating to current practice, barriers and incentives; which was used to code transcripts in QSR NVivo software. Two researchers independently coded each of the transcripts. Coding discrepancies were resolved through discussion between the two researchers, with any remaining disagreements resolved on the decision of a third member of the research team. An Aboriginal investigator on the research team (AC) provided insight on the narrative emerging from the qualitative analysis during the investigator meetings, and the results of the paper were discussed with 2 members of the Tackling Indigenous Smoking (TIS) team from the Aboriginal Health Council of SA (AHCSA) – which includes Aboriginal man Uncle Trevor Wingard - to understand how the findings fit with their lived experience in community. This information, alongside feedback from the advisory group, were incorporated in the discussion. For descriptive analysis of questionnaire data, Microsoft Excel was used.

### Ethics approvals

Ethics approvals were obtained from the ethics committees of The University of South Australia (39059), CALHN (04-19-851 (AHREC)) and the Aboriginal Health Council of South Australia (04-19-851).

## Results

### Participant characteristics

Interviews were conducted with *n* = 18 HPs; which included *n* = 10 doctors, *n* = 4 nursing staff, and *n* = 4 allied health professionals. Participants’ mean age was 43 years; 44% were female and 56% male. All participants worked with inpatients and most (76%) also provided outpatient services within the hospital (Table 1). Most had been treating Aboriginal patients throughout the majority (mean = 14 years) of their practicing experience (mean = 17 years). Most HPs reported that they discuss smoking cessation during consultations (89%). All but one participant (94%) had access to a smartphone or similar device during their workday. Only *n* = 17 HPs completed the questionnaire (Table 2).

**Table 1:** Characteristics and professional experience of interview participants (*n* = 18)

Characteristic	Overall sample (*n* = 18)
**Age, years**	
Mean	43.3
SD	14.1
**Gender**, ***n (%)***	
Male	10 (55.6)
Female	8 (44.4)
**Health profession category**, ***n (%)***	
Doctor	10 (55.6)
Nurse	4 (22.2)
Allied health professional	4 (22.2)
In which setting(s) do you treat Aboriginal patients? *, ***n (%)***	
Hospital	
Emergency Department	2 (11.1)
Inpatient	17 (94.4)
Outpatient	13 (72.2)
Primary care	2 (11.1)
Community care	4 (22.2)
Patient type^†^	
Paediatric	1 (9.1)
Adult	11 (100.0)
Geriatric	4 (36.4)
Location^‡^	
Metropolitan	8 (100.0)
Regional or rural	4 (50.0)
**Experience in health profession**, ***years***	
Mean	16.7
SD	12.8
**Experience treating Aboriginal patients**, ***years***	
Mean	14
SD	11.4
**Do you provide smoking cessation counselling during consultations?**, ***n (%)***	
Yes	
No	16 (88.9)
	2 (11.1)
**Do you have access to a smartphone or smart device?**, ***n (%)***	
Yes	17 (94.4)
No	1 (5.6)

*More than one category can be selected; ^†^missing data: *n* = 7; ^‡^missing data: *n* = 10

**Table 2:** HP experience and perspectives – questionnaire results (*n* = 17)

		Strongly agree	Agree	Unsure	Disagree	Strongly disagree
Statement/question		*n (%)*	*n (%)*	*n (%)*	*n (%)*	*n (%)*
I feel confident in my ability to adequately deliver smoking cessation advice/education to my Aboriginal patients		1 (5.9)	7 (41.2)	6 (35.3)	3 (17.6)	0 (0.0)
I have the support and resources to adequately manage Aboriginal patients who smoke		2 (11.8)	2 (11.8)	7 (41.2)	6 (35.3)	0 (0.0)
I understand what my role is within a multi-disciplinary team in the care and treatment of Aboriginal patients who smoke		3 (17.6)	13 (76.5)	1 (5.9)	0 (0.0)	0 (0.0)
I actively seek more information about new advances in smoking cessation		6 (35.3)	6 (35.3)	1 (5.9)	4 (23.5)	0 (0.0)
I think that smartphone based training would be beneficial to myself and other health professionals who deliver quit smoking education to Aboriginal patients		5 (29.4)	9 (52.9)	3 (17.6)	0 (0.0)	0 (0.0)
I believe smartphone technology would be useful for Aboriginal people to learn about smoking cessation		6 (35.3)	5 (29.4)	5 (29.4)	1 (5.9)	0 (0.0)
		Very good *n (%)*	Good *n (%)*	Fair *n (%)*	Poor *n (%)*	Very poor *n (%)*
In your experience, what is the standard of delivery of quit smoking advice and education to Aboriginal patients in South Australia?		0 (0.0)	2 (11.8)	8 (47.1)	7 (41.2)	0 (0.0)

### Current practice, resources, and technology use

Although all HPs supported the notion that every HP has a responsibility to discuss smoking cessation with patients, most rated the standard of smoking cessation advice currently provided to Aboriginal patients as only fair or poor (*n* = 15 of 17, 88%). Only 12% (*n* = 2 of 17) of participants felt that existing support and resources enabled them to adequately support Aboriginal patients who smoke. Selected questionnaire results are shown in Table 2.

Almost all HPs reported using either a smartphone or computer to access work-related information throughout the day (*n* = 17, 94%). Examples of work-related smartphone use included communication with colleagues, accessing contact details of other HPs, and using apps for purposes including medication information and clinical management guidance.

### Qualitative analysis

Of the 7 constructs within the TFA, 5 were coded in participant interviews. A summary of findings table with examples of identified themes can be found in Table 3.

**Table 3:** Summary of findings from qualitative analysis, with examples

TFA construct	Findings
Affective attitude	• Need for smoking cessation resources • Need for resources to be culturally tailored • Content and delivery method engaging for patients • Smartphone usage widespread and a popular way to access information – particularly for young people • The workbook provides an alternative way to access the information • Enables self-efficacy for patients to access information • Further development of technology to maximise ease of use • For health professionals, quick access to clear, brief, evidence-based information preferred • Alternative resource types should be offered to suit the work environment
Perceived effectiveness	• Content and delivery method engaging, relatable and culturally appropriate for Aboriginal patients • Valuable reminder for health professionals to discuss smoking cessation • Guide to systematically and effectively sharing key information with patients
Opportunity cost	• Other health priorities to be discussed within limited consultation time
Burden	• Time required to discuss smoking cessation with patients
Ethicality	• Access to technology a potential barrier for patients (particularly in older age group and potentially in remote areas)

#### Affective attitude

Affective attitude refers to the way a participant feels about an intervention [39], and can be separated into feelings experienced before and after exposure to the intervention (i.e. anticipated and experienced affective attitude).

#### Anticipated affective attitude

Overall, participants were supportive of the development of both sets of resources (for Aboriginal people who smoke and HPs), recognising the need for new and improved, culturally tailored smoking cessation support. Most felt that the use of technology, in the form of digital content accessed through a smartphone, would be well received by Aboriginal patients. Many HPs had observed that smartphone usage among Aboriginal patients was widespread even in remote communities, as one participant described that most patients *“…have electricity and reception. We send them appointment reminders [by] smartphone…”* – Doctor, male, aged 63. Many suggested that patients would find smartphone apps more fun and engaging than printed information: *“….like all of us, they’ve got their phones in their hands a lot of the time…. like playing games, that kind of stuff….that’s something that is easy to use, that is interactive and helpful…”* – Pharmacist, female, aged 29. Another perceived benefit for patients was that using a smartphone app would enhance their self-efficacy/self-confidence to access the specific information they need, at the time and place of their choosing. There was, however, a general perception that older patients would be less likely to engage with technology, so it would also be valuable to offer a printed workbook that could serve as a stand-alone resource containing all of the relevant information.

A preferred feature of digital information for HP resources was the ‘self-service’ approach, i.e. the ability to quickly and easily access the specific information required.

#### Experienced affective attitude

Patient resources: After viewing and interacting with the patient resources, the feedback provided was generally positive; with reports of perceived appropriateness and suitability for patients. The final patient resources shown to participants (V3) contained a mix of educational and practical information as well as fun and engaging activities that could be used for the purpose of distraction during a quit attempt, and this final content was very well received. The information also highlighted the benefits of quitting (in addition to the negative health effects), as this positive messaging was considered to be sometimes lacking in hospitals: *“it’s always in the context of ‘you have bronchiectasis exacerbation or pneumonia….’, but often we omit the positives as in ‘you have more money’”* – Respiratory registrar, female, aged 29. Most HPs (94%) agreed or strongly agreed that they would recommend the technology to their patients. Some suggested that female patients who smoke would be more likely than males to engage with the resources.

HP resources: Overall, attitudes towards the AR experience of V1 resources were quite positive, with most participants finding it engaging, novel and exciting. V2 and V3 resources received positive feedback from all participants, who highlighted the benefits of having easily accessible information, for example: *“I actually don’t have much access to information in hand when I’m doing the clinics so having anything in place certainly would be quite helpful*” – Basic training physician, male, aged 32. Characteristics of the resources that received favourable comments included useful and relevant content, attractive presentation including Aboriginal artwork, and the clear and brief presentation of the information, using bullet points. Different benefits and drawbacks were identified for each type of resource. The penholder (V2) was considered valuable in terms of providing “information at your fingertips”. However, some participants expressed concerns about whether they would be permitted to use penholders on shared desks due to their hospitals’ ‘paper-free’ desk policies, which led to the development of the poster (V3). On the other hand, a potential drawback of the poster was that *“an A3 sheet is something that there’s a lot of around the place and could it potentially be just lost amongst one of many, many posters on a wall”* – Doctor, male, aged 31.

#### Perceived effectiveness

This construct refers to “the extent to which the intervention is perceived as likely to achieve its purpose”^37^, and can also be separated into anticipated and experienced categories.

#### Anticipated perceived effectiveness

When asked about the potential for AR technology to deliver health information, most participants felt that it was likely to be effective. Some described how smartphones were already being used successfully in other areas of healthcare. AR was considered likely to be more engaging and therefore better able to convey the message than other digital formats, due to the novelty of AR conveying health information: “*it’s good to have resources that are easy to engage with and people do like, you know, fun things. They like songs, engaging, dancing, those sort of physical sorts of things*.” – Physician, male, aged 64. The popularity of smartphones was believed to apply to young people more than older people.

One participant reflected on the potential value of a resource that reminds HPs to raise the issue of smoking cessation. Although recognised as an important priority, it was suggested that in the reality of busy hospital practice, discussion about smoking can sometimes be overlooked: *“But then [staff are] really tired, four o’clock in the morning… then sometimes you forget. It shouldn’t happen, but it does happen in real life. It happens. And then the opportunity is missed.”-* Doctor, male, aged 63.

### Experienced perceived effectiveness

#### Patient resources

After viewing and experiencing the patient resources, most participants thought that the workbook and associated app had the potential to improve the quality of smoking cessation counselling during consultations: “*maybe having a resource to be able to show the patient how smoking damages the body and that sort of thing, you know, visually it might be helpful to engage them in that quitting discussion”* – Doctor, male, aged 34. Some HPs suggested that it would give them confidence to be able to provide the important information to patients in a more comprehensive and systematic way, and to ensure that none of the key information is missed. Cultural tailoring of health education resources was another cited expected benefit that could increase the effectiveness of resources. One participant had observed that communication with Aboriginal patients was considerably improved by involving an Aboriginal Liaison officer; but as these staff are not able to be present during every interaction, it was suggested that showing videos of Aboriginal people sharing their stories would be an alternative way to make the information relatable.

It was also recognised that smoking cessation is an ongoing process and, while the resources may encourage and support people who smoke to take the first step, the HP role in continuing and maintaining smoking cessation was less clear, as described in the following comment: “*- as soon as you leave hospital, life becomes more complicated and busier and everything*” –Cardiologist, male, aged 35. On the other hand, it was acknowledged that some of the included content did have the potential to assist people further along the quitting journey: *“the best bit of this I think, is all the cravings and distractions and how do we help? Which is clearly somebody that’s already, sort of, started the journey.”* – Oncologist, male, 57.

#### PHP resources

All participants felt that the HP resources would be effective in improving their skills and confidence, as illustrated by the following comment: “*if there’s everything here….when [they] ask [a] question, then you just answer straight away. The people will be more trusting.”* – CN, female, aged 47. Most felt that printed information would be a more effective way to convey the information to HPs as it was quicker and easier to find the required information, for example: “*I would probably prefer that to be in written format so that …[I’ll] be able to go to that precise part that I have forgotten or need more information about*” – Doctor, male, aged 34. Several HPs said that, to increase their skills and confidence, they would benefit from more in-depth information about the ‘Ask, Advise, Help’ counselling technique. One suggestion was to distribute the printed resources in conjunction with a training session, then the printed information could serve as a reminder, as one participant said: “*I think [the printed resources] could work quite well, but there has to be substance underneath it*” – Nurse consultant, female, aged 64.

#### Opportunity cost

Opportunity cost is defined as “the extent to which benefits, profits, or values must be given up to engage in the intervention”^37^. The main opportunity cost identified was the need to address other healthcare priorities in the context of limited consultation time. One HP described the experience of only having to prioritise in terms of the information discussed: “*in the level of interaction that I’m having with the patients when they’re here….[other topics are] certainly not high on the agenda*.”- Cardiologist, female, aged 57.

V1 HP resources were not well received and were considered (a) too cumbersome (the need to install and open an app then scan the postcard), and (b) time-consuming. The following was typical of the feedback: “*I don’t know whether for a professional, you need that same augmented thing because you could, if you have an app that you could use to potentially get information that could also have links to video links, to educational messages from experts around things that you could use, I find it a little bit….unnecessary to actually have to hold the phone….and you need to get [the alignment] right*” – Surgical oncologist, male, aged 60.

### Burden

Burden refers to the “perceived amount of effort that is required to participate in the intervention [39]”.

Patient resources: Though usability of the patient resources was rated very positively overall, with 88% (16 of 18) of HP participants responding that the app was easy or very easy to use, some suggested that lack of familiarity with AR and the method of scanning images and/or pages could be a barrier. QR codes were suggested as an alternative with which community members might be more familiar.

HP resources: All but one participant (94%) agreed or strongly agreed that it easy to use the HP resources, including AR functionality. The main burden identified related to the initial set of HP resources demonstrated in the first round of interviews (V1), as per Opportunity Cost above.

### Ethicality

Ethicality is “the extent to which the intervention has good fit with an individual’s value system”^37^. Concerns were raised about the suitability of the technology for certain demographic groups, mainly for the elderly, but also for those with limited access to technology or stable internet access – suggesting that the intervention may not be appropriate or accessible for all. For example, one participant commented that “*some of the younger generation patients would probably use it… if they’ve got the phone access and also there’s no language barrier, yeah*” – Pharmacist, female, aged 30.

## Discussion

In a mixed methods study of 18 HPs from South Australia, researchers aimed to iteratively co-design resources for both Aboriginal people who smoke, and their healthcare providers, via interviews and questionnaires. A perceived lack of resources for Aboriginal people who smoke and their healthcare providers was identified, however, the majority of HPs (89%) reported that they discussed smoking cessation during consultations. This is in contrast to existing literature, which suggests that HPs are unable to provide smoking cessation advice – particularly to Aboriginal patients - owing to lack of time, knowledge, skills, and confidence [11–15]. This is also contrary to the experience of the TIS team, suggesting that 89% was quite high. Aboriginal Health Workers (AHWs) in remote communities often struggle to ask their patients about smoking, especially their elder patients, for fear of being seen as “challenging” their elders, or out of self-consciousness as many AHWs are themselves smokers. Many interviewed HPs were not aware of any culturally appropriate resources that were available in South Australia, despite the fact that these resources do exist. Issues with governance structures within AHCSA including lack of media representation, as well as unclear navigation pathways through health services mean that HPs, and in turn Aboriginal patients, are unaware of the resources they need. Clarification of referral pathways from AHWs to GPs for NRT prescription or to Aboriginal Quitline counsellors would reduce the time and psychological burdens on AHWs, and facilitate access to appropriate services for patients.

Factors influencing the acceptability of resources were identified across 5 domains of the TFA (Table 3). Participants expressed broad acceptability for the sets of resources designed to support (i) Aboriginal people who smoke, and (ii) the healthcare providers who treat them. Unsurprisingly, acceptability was highest for the final sets of resources that were developed with participants through the iterative co-design process. The co-design process aims to ensure a high quality output which is meaningful to end-users [30]. This process has been undertaken in a multitude of healthcare settings, as summarised in a rapid overview of reviews from 2020 [29]. This overview concluded that co-design can benefit all involved; engagement with end-users at these early stages ensures that the resulting intervention is relevant, user-friendly, and specific, and existing data suggests that co-design also benefits the researchers and practitioners involved, along with research processes and research outcomes. A similar process was undertaken in the development of educational resources for pregnant Aboriginal women in the Indigenous Counselling and Nicotine (ICAN) QUIT in Pregnancy project, which utilised a participatory action research approach [40, 41].The co-design process raised new ideas about how to develop resources that were most acceptable and useable for the target audience, and authors concluded that “*Sharing the process of resource development and learning can better inform future health promotion and interventions*”.

For the patient resources, affective attitudes were favourable in terms of the resources’ perceived cultural appropriateness, engaging and relatable style and delivery method, with evidence-based information that is clear, concise and presented using lay language. A perceived benefit for patients was having access to comprehensive information in an easily accessible format (with both video and text options) that could help to empower patients and promote agency, factors that have been identified as important for increasing inclusion of Aboriginal Australians in health messaging [6]. This was supported by the TIS team, who mentioned the language barriers in the APY lands as a significant barrier to health resource development and overall health communication. Overall, the use of AR was considered to have great potential for delivering the quit smoking messages to patients, and this is consistent with previous research suggesting that a technology-based solution has the potential to reduce inequality in Aboriginal health [33]. The perceived effectiveness was positively influenced by the resources’ potential to address key barriers to Aboriginal people accessing health information. Culturally tailored content and language together with visual images (printed) and audio visual content (digital) could address the need to reach people with poor health literacy [21]. For those in remote areas, access to user-friendly information through their own smartphone and/or workbook also has the potential to address the problem of limited accessibility to information and geographic reach for health services [22]. However, as identified by participants as a potential barrier related to the ethicality domain, there is currently a large gap in access to digital resources between individuals living in remote First Nations communities and those living in other areas. A 2023 ‘Measuring the Digital Gap’ report identifying that the former are the most digitally excluded people in Australia; with 45.9% of survey respondents rated as highly excluded, compared with only 9.2% of other Australians [42]. When discussing the potential benefits of delivering health information via smartphone, most participants mentioned age as an important factor, suggesting that it would be less appropriate and acceptable for the older age group. Some HPs also suggested that women were more likely than men to engage with the resources. The TIS team supported this suggestion, describing women as leaders within the community and making reference to the matriarchal structures often seen within Aboriginal communities [43]. This gap also speaks to a larger issue in Australian society, in which men often feel unable to seek support or express vulnerabilities [44, 45]. Issues identified throughout this study highlight the need for decolonisation of research and healthcare methodologies. The inclusion of Aboriginal models of health care to help address Aboriginal health concerns, due to the limitations of Western models of care in general, and specifically for Aboriginal communities, is integral to the health and wellbeing of Aboriginal Australians [8]. In relation to the resources developed for this study, it was suggested that further development was needed to improve ease of use.

Regarding HP resources, affective attitudes indicated a preference for clear and brief advice on evidence-based approaches delivered in text form, to increase HP skills, knowledge and confidence. The initial iteration – postcards with AR-activated content – was considered to be easy to use, however it was considered to be too cumbersome for HPs to use in practice. This is unsurprising, as the major barrier to HPs providing smoking cessation advice was time constraints and the competing demands of other aspects of patient care. The brevity of the recommended ‘Ask, Advise, Help’ technique was therefore an important feature for HPs who feel they lack the time to provide in-depth counselling [35]. The final set of HP resources – the poster – was considered by most participants to be valuable as a reminder to “have the conversation” and to improve their knowledge and confidence. Increasing the likelihood of HPs discussing smoking with patients could have huge benefits for reducing smoking rates [46]. Indeed, smoking cessation has been identified as one of the most cost-effective treatments that HPs can provide [47]. Identified barriers such as a paper-free desk policy highlighted the importance of offering a delivery method suitable for the work environment and led to the development of posters (V3) in addition to penholders (V2) developed earlier in the project. Participants identified that the printed resources would make a valuable addition to more in-depth, face-to-face training sessions – which has been shown to be beneficial in improving smoking cessation rates in both primary care and pharmacy settings, as well as specifically in Aboriginal populations [9, 12, 17]. This has informed the next phase of work in this space; with a culturally-aware smoking cessation training program for HPs being evaluated through a cluster-RCT in South Australian hospitals (manuscript under review).

### Limitations

HPs were not asked to disclose their ethnicity in the demographic questionnaire, and while we understand through interview transcripts that some participants were Aboriginal, it is unclear how many of the participants were Aboriginal. While the HP resources were originally co-designed with an Aboriginal advisory group (which included Aboriginal HPs), in order to more fully understand the cultural appropriateness of the HP resources for Aboriginal Australians who smoke, it is essential to include the opinions of Aboriginal HPs. Acceptability of the patient resources by Aboriginal people has been explored in a separate paper [48] (manuscript under review).

## Conclusions

In a mixed methods co-design study of 18 HPs from SA, participants were very amenable to providing the AR technology-enhanced smoking cessation workbook to their patients, as it addresses the need for resources that are culturally appropriate, engaging and relatable, with evidence-based information that is clear, concise and presented using lay language. The HP resources based on brief counselling techniques were considered a helpful reminder to prompt a culturally-appropriate, smoking cessation conversation with Aboriginal patients, when presented in an appropriate format for time-poor HPs. These resources may serve as a useful adjunct to more in-depth HP training sessions. This study also highlighted gaps in health service referral pathways which prevent Aboriginal people who smoke from accessing culturally appropriate and effective resources to help them quit smoking. High acceptability of the information and delivery method indicate potential for AR-delivered resources for both patients and HPs; ultimately providing an additional pathway to increase support for Aboriginal patients to quit smoking.

## Supplementary Information

Below is the link to the electronic supplementary material.

Supplementary Material 1

## Data Availability

The de-identified data we analysed are not publicly available, but requests to the corresponding author for the data will be considered on a case-by-case basis.

## Abbreviations

AR: Augmented Reality
CONSIDER: CONSolIDated critERia for strengthening the reporting of health research involving Indigenous Peoples statement
HP: Health Professional
RACGP: Royal Australian College of General Practitioners
TFA: Theoretical Framework of Acceptability
