# Supplementary material for: Developing novel smoking cessation resources for Aboriginal people who smoke tobacco and their healthcare providers: a mixed methods study
Source: BMC Health Serv Res. 2025 Nov 28;25:1610. doi: 10.1186/s12913-025-13568-x (PMC12723829; doi:10.1186/s12913-025-13568-x)
Supplement: Supplementary file 1 — Supplementary Material 1 [file 12913_2025_13568_MOESM1_ESM.docx]

**Appendix 1: CONSIDER checklist**

| Governance | | |
| --- | --- | --- |
| 1 | Describe partnership agreements between the research institution and Indigenous-governing organization for the research, (e.g., Informal agreements through to MOU (Memorandum of Understanding) or MOA (Memorandum of Agreement)). | Partnership agreements were approved between SA Health Research Committee (CALHN – QEH, RAH), UniSA, Adelaide University and the Aboriginal Health Research Ethics Committee (AHREC). |
| 2 | Describe accountability and review mechanisms within the partnership agreement that addresses harm minimization. | The resources were developed under the guidance of an Aboriginal Advisory group to ensure cultural sensitivity and appropriateness.  A protocol was developed and approved by ethics to minimise risk of harm and to ensure participants expressing distress are cared for.  Between the research team, the Investigator team, and the advisory group, there was regular correspondence via email, as well as via regular meetings. When an issue was raised during the project, it was initially raised with the advisory board via email. At the regular meetings, these issues were discussed and any action to be taken was discussed with the group. Follow-up emails were sent to those not in attendance so that everyone was aware of the decisions made. |
| 3 | Specify how the research partnership agreement includes protection of Indigenous intellectual property and knowledge arising from the research, including financial and intellectual benefits generated (e.g., development of traditional medicines for commercial purposes or supporting the Indigenous community to develop commercialization proposals generated from the research). | Section I of the approved ethics application includes information about data ownership. “Aboriginal people and their communities own, protect and control their own data. As researchers we are seeking through a process of informed consent to access and use this data to ultimately improve health outcomes for Aboriginal South Australians. The data collected and analysed and intellectual property created in fulfilment of the aims of this study will be owned by University of South Australia and will be shared with the community through publication and appropriate public engagement.”  This project aims to build capacity of the South Australian health care system to better meet the needs of Aboriginal people prioritising evidence based, best-practice care. The idea for this project is driven by community need through consultation with local Aboriginal people, who believe that both smoking cessation and the use of technology may be a useful resource.  We have also commissioned local artwork by an Aboriginal artist to use for the resources and we showcase and promote local Aboriginal talent, culture and environment for the videos produced. |
| Prioritization | | |
| 4 | Explain how the research aims emerged from priorities identified by either Indigenous stakeholders, governing bodies, funders, non-government organization(s), stakeholders, consumers, and empirical evidence | This project has come about following over a decade of previous research into smoking cessation among Aboriginal Australians undertaken by A/Prof Carson-Chahhoud. Following one-on-one interviews with Aboriginal smokers, ex-smokers, non-smokers, key community stakeholders, Elders and health workers, the consensus was that something new in this space was needed to make a real difference. Informal conversations with over 100 Aboriginal Elders, community members and key stakeholders has also taken place over the past decade. |
| Relationships (Indigenous stakeholders/participants and research team) | | |
| 5 | Specify measures that adhere and honour Indigenous ethical guidelines, processes, and approvals for all relevant Indigenous stakeholders, recognizing that multiple Indigenous partners may be involved, e.g., Indigenous ethics committee approval, regional/national ethics approval processes. | Ethics approval received from Aboriginal Health Research Ethics Committee (AHREC) |
| 6 | Report how Indigenous stakeholders were involved in the research processes (i.e., research design, funding, implementation, analysis, dissemination/recruitment). | In addition to Section 4 above, the resources were developed under the guidance of an Aboriginal Advisory group to ensure cultural sensitivity and appropriateness.  The Advisory Group’s primary duty and function is to oversee the conduct of the research project, and to:   - Provide expert advice as to the project methodology including planning, development, implementation and evaluation of the project and to amend the project plan as required based on evidence or outcomes as they come to hand. - Review the project management plan and to inform this process as required. - Contribute to, guide and oversee the research related activities of the project. - Ensure that the outcomes and outputs of the project are relevant, meaningful and are translated in policy documents that then inform or translate into clinical practice, system changes and /or practical interventions.   Membership comprises of Aboriginal representatives from the community, spanning a range of ages and positions within the community.  The Tackling Indigenous Smoking team from the Aboriginal Health Council of South Australia (which includes Uncle Trevor Wingard) provided their insight on the results; comparing the findings to their own lived experience working with Aboriginal communities rurally and remotely across South Australia. |
| 7 | Describe the expertise of the research team in Indigenous health and research. | **Kristin Carson-Chahhoud:** an experienced Aboriginal health researcher, importantly, Aboriginal community engagement on the topic of tech-based health messaging began in 2010, when CIA consulted over 100 Aboriginal Elders, key stakeholders, & community members as part of her PhD. Her PhD developed new evidence for the treatment of tobacco addiction within hospital settings, and for Indigenous populations globally.  **Gillian Gould**: a Professor in Health Equity Southern Cross University (SCU), whose research career has focussed on improving tobacco smoking risks for Indigenous Australians over the past 20 years. Gould works closely with Aboriginal communities and Aboriginal Medical Services, and has built up trusted relationships; she regularly participates in Aboriginal events. Over 20 years she co-developed community-based strategies to tackle Aboriginal smoking, led a 2-year regional participatory action research project as part of the Indigenous Tobacco Control Initiative and collaboratively led the co-design of an Aboriginal intervention for smoking among Aboriginal pregnant women (ICAN QUIT in Pregnancy/SISTAQUIT/iSISTAQUIT (Supporting Indigenous Smokers To Assist Quitting)) to scale-up in over 100 services.  **Haydyn Bromley**: executive director of Bookabee Australia. Haydyn has 35+ years’ experience in education and training, delivering Aboriginal Cultural Sensitivity and Respect training, Cultural Awareness and capability training.  **Alwin Chong**: a Wakamin man from North Queensland, holding an Associate Professor position at the University of South Australia. He leads the Positive Futures Research Collaboration unit that has been established to conduct rigorous research into Aboriginal child protection. He established the Young Dads Program which engages with Aboriginal male youth to help them heal and promote living and parenthood skills. |
| Methodologies | | |
| 8 | Describe the methodological approach of the research including a rationale of methods used and implication for Indigenous stakeholders, e.g., privacy and confidentiality (individual and collective) | As per Methods section: A mixed methods triangulation research design^31^ was utilised.    Within the AHREC application process, the Privacy Legislation Matters checklist was completed to ensure adherence to the Privacy Act. This was submitted to and approved by AHREC.  The AHREC application (as well as the associated study protocol) defined the data confidentiality plan, and this information was also included in the participant information forms. |
| 9 | Describe how the research methodology incorporated consideration of the physical, social, economic and cultural environment of the participants and prospective participants. (e.g., impacts of colonization, racism, and social justice). As well as Indigenous worldviews. | There was direct engagement with local community members in all stages of the research project: from study inception and research design, through to data analysis and interpretation. |
| Participation | | |
| 10 | Specify how individual and collective consent was sought to conduct future analysis on collected samples and data (e.g., additional secondary analyses; third-parties accessing samples (genetic, tissue, blood) for further analyses). | N/A |
| 11 | Described how the resource demands (current and future) placed on Indigenous participants and communities involved in the research were identified and agreed upon including any resourcing for participation, knowledge, and expertise | N/A |
| 12 | Specify how biological tissue and other samples including data were stored, explaining the processes of removal from traditional lands, if done, and of disposal. | N/A |
| Capacity | | |
| 13 | Explain how the research supported the development and maintenance of Indigenous research capacity (e.g., specific funding of Indigenous researchers). | Aboriginal members of the study team had an important role in the project design, along with analysis and interpretation of the outcomes of the research project itself. They will also contribute to and receive authorship on academic outputs from the project. Following this, they will join other members in the team to develop a strategy for translation of research outcomes and resources into practice so that the benefits may be accessed by a wider range of people from the community. |
| 14 | Discuss how the research team undertook professional development opportunities to develop the capacity to partner with Indigenous stakeholders? | Any training required in the conduct of research methodology will be provided by the expert research team, led by A/Prof Carson-Chahhoud. This will include training in the conduct and analysis of qualitative research. Training in design techniques, augmented reality technology and cultural appropriateness/safety will be provided by Prof Ian Gwilt (Professor of Design at UniSA), Dr Ross Smith (Co-Director of the Wearable Computer Laboratory UniSA) and Mr Haydyn Bromley (Owner of Bookabee Australia Pty. Ltd.) respectively. |
| Analysis and interpretation | | |
| 15 | Specify how the research analysis and reporting supported critical inquiry and a strength-based approach that was inclusive of Indigenous values. | Aboriginal investigators on the research team provided insight on the narrative of the results during the investigator meetings; this, alongside feedback from the advisory group, were incorporated in this analysis.  The Tackling Indigenous Smoking team from the Aboriginal Health Council of South Australia (which includes Uncle Trevor Wingard) provided their insight on the results; comparing the findings to their own lived experience working with Aboriginal communities rurally and remotely across South Australia. |
| Dissemination | | |
| 16 | Describe the dissemination of the research findings to relevant Indigenous governing bodies and peoples. | As per the study protocol and ethics applications: a memo will be circulated to relevant Aboriginal health organisations and community organisations and an offer extended to have a member of the research team come out to give a presentation explaining the research process, outcomes and the potential application of the results to them and their community. |
| 17 | Discuss the process for knowledge translation and implementation to support Indigenous advancement (e.g., research capacity, policy, investment). | If proven to be acceptable, the resources will be delivered in the hospital setting, with training of hospital staff in both smoking cessation for Aboriginal patients, as well as cultural awareness training delivered by Haydyn Bromley, that should help their overall provision of care for Aboriginal Australians beyond the scope of this tobacco project. In future, we intend to make the resources freely available via the Apple store and Google Play after project completion. Likewise, the printed resources will be freely available and circulated via Cancer Council and SA Health after project completion. |

**Appendix 1: Questionnaire**

**PART B: SMOKING CESSATION EDUCATION IN ABORIGINAL HEALTH** (prior to

interview) *Please circle one option per question:*

**PART A: BACKGROUND**

1. Name:
2. **Age: 3. Gender:** Male / Female
3. **Occupation: 5. Qualifications *(Please list all):***
4. **In what setting/s do you treat Aboriginal South Australians? *(Tick all that apply)***
   - Emergency department □ Paediatric
   - Inpatient □ Adult
   - Outpatient □ Geriatric
   - Primary care □ Metropolitan
   - Community □ Regional / Rural
   - Paramedical
   - Other. Please specify:
5. How many years have you practiced in your profession? years months
6. How many years have you been treating Aboriginal patients? years months
7. Is education on smoking cessation provided during your consultations? YES / NO
8. Do you have access to a smartphone or other smart device/s? YES / NO

| I feel confident in my ability to adequately deliver smoking cessation advice/education to my Aboriginal patients | Strongly agree | Agree | Unsure | Disagree | Strongly disagree |
| --- | --- | --- | --- | --- | --- |
| I have the support and resources to adequately manage Aboriginal patients who smoke | Strongly agree | Agree | Unsure | Disagree | Strongly disagree |
| I understand what my role is within a multi-disciplinary  team in the care and treatment of Aboriginal patients who smoke | Strongly agree | Agree | Unsure | Disagree | Strongly disagree |
| I actively seek more information about new advances in smoking cessation | Strongly agree | Agree | Unsure | Disagree | Strongly disagree |
| I think that smartphone based training would be beneficial to myself and other health professionals who  deliver quit smoking education to Aboriginal patients | Strongly agree | Agree | Unsure | Disagree | Strongly disagree |
| I believe smartphone technology would be useful for Aboriginal people to learn about smoking cessation | Strongly agree | Agree | Unsure | Disagree | Strongly disagree |
| In your experience, what is the standard of delivery of quit smoking advice and education to Aboriginal patients in South Australia? | Very poor | Poor | Fair | Good | Very good |

# Turn page to complete PART C & D of this questionnaire AFTER the interview.

**PART C: APPLICATION AND USE** (after interview)

*Please circle one option per question:*

| I can use these resources including augmented reality functionality | Strongly agree | Agree | Unsure | Disagree | Strongly disagree |
| --- | --- | --- | --- | --- | --- |
| I think this technology could improve my ability to deliver best-practice advice to Aboriginal smokers | Strongly agree | Agree | Unsure | Disagree | Strongly disagree |
| I think this technology could improve smoking rates in the Aboriginal Community of South Australia | Strongly agree | Agree | Unsure | Disagree | Strongly disagree |
| I would use this program of resources frequently | Strongly agree | Agree | Unsure | Disagree | Strongly disagree |
| I would recommend this technology to my colleagues | Strongly agree | Agree | Unsure | Disagree | Strongly disagree |
| I would recommend this technology to my patients | Strongly agree | Agree | Unsure | Disagree | Strongly disagree |
| I think this technology can be used in other areas of health education for Aboriginal patients | Strongly agree | Agree | Unsure | Disagree | Strongly disagree |

**PART D: SOFTWARE REVIEW** (after interview): *Please one option per question:*

| How easy is it to navigate (move from one feature to another) through the App? | Very difficult | Difficult | Neutral | Easy | Very easy |
| --- | --- | --- | --- | --- | --- |
| How easy is it to **learn** how to use the App and features? | Very difficult | Difficult | Neutral | Easy | Very easy |
| How easy is it to **use** the App and features? | Very difficult | Difficult | Neutral | Easy | Very easy |
| How attractive is visual design (fonts and colours)? | Not  attractive at all | Not attractive | Fair | Attractive | Very attractive |
| Does the App appear well-organised? | Not well- organised at all | Not well- organised | Neutral | Well- organised | Very well- organised |
| Are the sizes of the fonts/buttons/videos appropriate? | Not appropriate at all | Not appropriate | Neutral | Appropriate | Very appropriate |
| Is the content presentation interesting? | Not interesting at all | Not interesting | Neutral | Interesting | Very interesting |
| What did you think about the quality of the AR? | Very poor | Poor | Fair | Good | Very good |
| Is the App irritating? | Very Irritating | Yes | Neutral | No | Not Irritating at  all |
| How interesting is the App? | Not interesting at all | Not interesting | Neutral | Interesting | Very interesting |
| What do you think about the accuracy of the information? | Very poor | Poor | Fair | Good | Very good |
| What do you think about the presentation of the information? | Very poor | Poor | Fair | Good | Very good |
| How would you rate the sufficiency of the information? | Very poor | Poor | Fair | Good | Very good |
| Is the intended use and purpose of the App clear? | Very unclear | Unclear | Neutral | Clear | Very clear |

*Appendix*

**Appendix 3a: Version 2 HP resource - penholder**

**
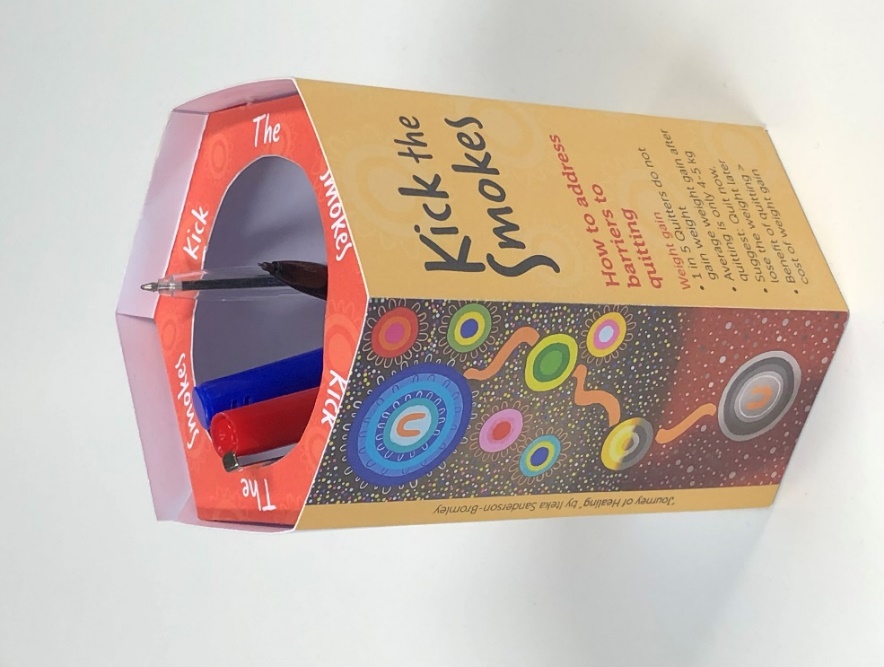
**

**Appendix 3b: Version 3 HP resource –poster**

**
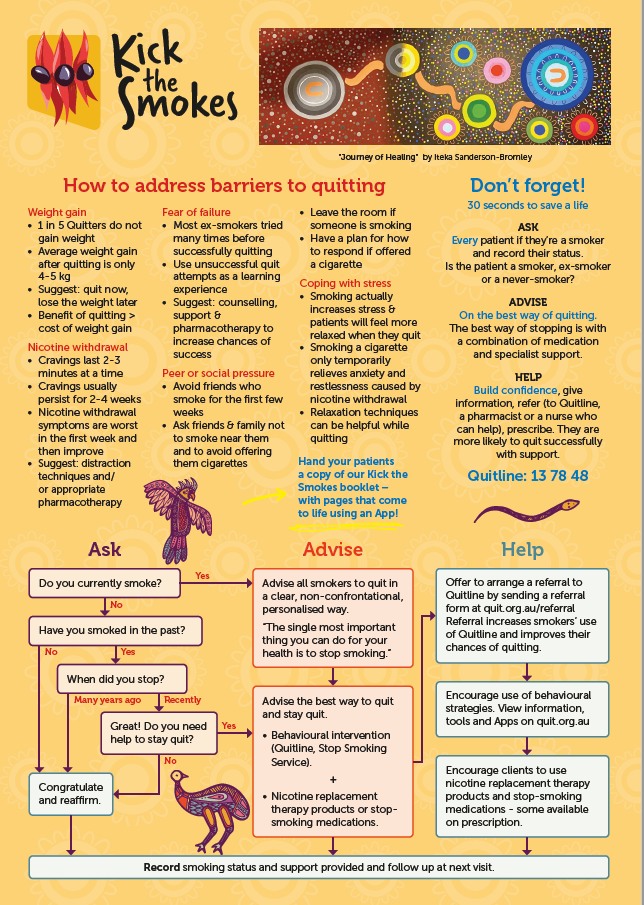
**
